# Multiphase Biopolymers Enriched with Suberin Extraction Waste: Impact on Properties and Sustainable Development

**DOI:** 10.3390/ma17225472

**Published:** 2024-11-09

**Authors:** Anita Wronka, Grzegorz Kowaluk

**Affiliations:** Institute of Wood Science and Furniture, Warsaw University of Life Sciences—SGGW, Nowoursynowska St. 159, 02-776 Warsaw, Poland

**Keywords:** PLA, starch, biopolymer, circular economy, recycling, SAR, wastes

## Abstract

This manuscript explores the development of sustainable biopolymer composites using suberin extraction waste, specifically suberinic acid residues (SAR), as a 10% (*w*/*w*) reinforcing additive in polylactide (PLA) and thermoplastic starch–polylactide blends (M30). The materials were subjected to a detailed analysis using thermogravimetric analysis (TGA), differential scanning calorimetry (DSC), and dynamic mechanical analysis (DMA) to assess their thermal, mechanical, and structural properties. The study confirmed the amorphous nature of the biopolymers and highlighted how SAR significantly influences their degradation behavior and thermal stability. M30 exhibited a multi-step degradation process with an initial decomposition temperature (T5%) of 207.2 °C, while PLA showed a higher thermal resistance with decomposition starting at 263.1 °C. Mechanical performance was assessed through storage modulus (E′) measurements, showing reductions with increasing temperature for both materials. The research provides insights into the potential application of SAR-enriched biopolymers in sustainable material development, aligning with circular economy principles. These findings not only suggest that SAR incorporation could enhance the mechanical and thermal properties of biopolymers, but also confirm the effectiveness of the research in reassurance of the audience.

## 1. Introduction

Biopolymers represent a promising alternative to traditional plastics due to their biodegradability and their ability to be produced from renewable resources. Biopolymers can decompose naturally into safe components such as water and organic matter, making them essential for minimizing environmental impact [1,2]. Biopolymers are derived from plentiful and renewable origins, including plants, animals, and microorganisms [3,4]. Examples of such materials include polysaccharides like starch and cellulose, proteins like gelatin and collagen, and bioplastics such as polyhydroxyalkanoates (PHA) and polylactic acid (PLA) [5]. Unlike finite petrochemical resources, these sources are considered virtually limitless [3]. Among them, PLA and polycaprolactone (PCL) stand out for their mechanical properties and environmental benefits, making them widely used in various industries. Owing to their compatibility with biological systems and lack of toxicity, biopolymers find applications across multiple industries, including biomedical engineering, food packaging, and renewable energy solutions. However, their relatively low strength and limited functionality present challenges to fully realizing the potential of these materials [6,7]. Modifying biopolymers by adding natural substances, such as tree bark or wood fibers, or wood wastes from producing other wood materials, has become a subject of intensive research to improve their properties and introduce additional functionalities. This approach supports the circular economy concept and offers new possibilities for sustainable material development. 

PLA and other biopolymers are frequently used as materials in 3D printing; however, research consistently highlights the need to improve their mechanical properties, explore recycling options for polymers and natural waste, and address the growing demand for sustainable practices. When leveraging waste and recycled polymer composites to produce biodegradable materials, stakeholders are encouraged to adopt circular economy models and sustainable manufacturing practices [8]. 

A common biopolymer, polylactic acid (PLA) has many drawbacks, including poor mechanical and heat resistance. Different fillers are added to PLA to improve its qualities in order to overcome these problems. To enhance performance, various fillers are incorporated. For example, non-wood lignocellulosic fillers derived from bamboo—such as sawdust, Kraft-process fibers, and low-lignin fibers—are used to improve the mechanical strength and fiber distribution within the PLA matrix [9]. The combination of pistachio shell flour and poly(butylene succinate) grafted with maleic anhydride (PBS-g-MAH) increased mechanical and thermomechanical hardness and stiffness while maintaining thermal stability [10]. Lignocellulosic fillers can accelerate biopolymer biodegradation. Pecan nutshells, for instance, increased the swelling and removal of the polymer’s amorphous phase in PLA composites, promoting quicker biodegradation and possibly improving PLA’s crystallinity and thermal stability, particularly when paired with physical processes like thermal annealing and chemical changes. As a result, the overall thermal performance and heat deflection temperatures are enhanced [11]. Using lignocellulosic corn cob powder as a filler in a PHB (polyhydroxybutyrate)/PLA biopolymer matrix, eco-friendly polymer composites were developed, demonstrating the potential of agricultural leftovers to support the sustainable development of materials [12]. Sunflower hulls were used as fillers in polypropylene-based biocomposites and they were byproducts of seed stripping for biodiesel manufacturing. They undergo processing by injection molding and melt compounding, which enhances their mechanical qualities [13]. PHB co-polymers now have hemp and alfalfa particles added, which significantly improves stiffness and the heat deflection temperature while also strengthening flame-retardant attributes [14]. High-density polyethylene (HDPE) biocomposites were made using cotton stalk fillers, which can enhance tensile and flexural moduli. The best results are obtained at higher filler loadings [15]. PHB biocomposites were filled with cellulose and calcium carbonate. Because of aggregation, cellulose marginally reduces mechanical qualities; conversely, calcium carbonate increases heat stability and Young’s modulus [16]. When used as a PLA filler, spirulina preserves a high Young’s modulus and improves toughness and tensile strength, particularly when mechanically processed [17].

PLA’s crystallinity can be enhanced, and its semi-crystallization time is shortened by adding wood fibers, such as poplar fiber, which would increase the biopolymer’s heat stability and processing efficiency [18]. Nevertheless, adding sawdust and wood ashes lowered the PLA’s glass transition temperature and thermal stability, indicating that the kind and quantity of bark additive can significantly impact thermal characteristics [19]. PLA gains much better mechanical characteristics when Bael bark is added. Compared to pure PLA, a composite containing 15-weight percent Bael bark showed gains in tensile strength, compressive strength, and flexural strength of 39%, 33.8%, and 33.9%, respectively [20]. Bark additives can potentially improve the strength and durability of PLA-based materials, as demonstrated by wood leachate (WL) powder improving the PLA biocomposites’ mechanical properties [21]. Bark additives do not significantly impair the biodegradability of the biocomposites, as evidenced by the slight decrease in the biodegradation rate, but the continued high levels of disintegration in the compost media following the addition of wood particles to PLA/PHB blends still occur [22]. Biodegradability can be further influenced by adding natural fillers to PLA/PHB mixes, such as wood particles or biochar. For example, adding biochar to mixes of PLA and PHB causes the PLA to degrade more quickly during hydrolysis [23]. Similarly, wood particles in a PLA/PHB mix disintegrate significantly in compost but at a slower rate than in pure PLA. In general, PLA/PHB mixes, including natural fillers—such as wood particles or biochar—degrade more quickly than PLA or PHB alone. This results from the fillers’ synergistic effects and the biopolymers’ intrinsic biodegradability [22]. Natural fillers may improve biodegradability; however, their mechanical qualities may also be impacted. When isolated cellulose is added to PLA/ABS blends, for instance, the weight loss during biodegradation rises, but the tensile strength and elongation at breakage decrease [24]. 

Additives and plasticizers can improve PLA’s thermal stability, which can impact crystallinity and the glass transition temperature (Tg) [25,26]. The melting point of PHB is around 170–180 °C. To prevent fast thermal deterioration, PHB should be treated slightly over its melting point, usually 180–190 °C [27,28].

The biodegradation rate of PLA depends on several factors, including its composition, whether it contains additives that can accelerate the process, or the conditions under which it is stored. Compared to natural weathering, PLA breaks down more quickly when composting settings are used. Complete biodegradation can happen in 30 days under actual composting circumstances, although lab models reveal slower rates [29]. Significant weight loss was noted over a six-month period, indicating that landfill conditions also encourage deterioration more quickly than natural weathering [30]. When some bacteria, including *Pseudomonas geniculate* WS3, are used with soytone, PLA biodegradation is greatly accelerated. PLA film fragmentation and significant weight loss occur within 20 days of this combo [31]. Furthermore, high-molecular-weight PLA films may be broken down by a group of bacteria that includes *Nocardioides zeae* and *Stenotrophomonas pavanii*. In 35 days, this degradation results in a 61% decrease in molecular weight and a 9.68% weight loss [32]. Different degradation rates are displayed by modified PLA products, such as those mixed with polybutylene succinate (PBS). The PBS-modified PLA breaks down more quickly at first, but in the later phases of composting, pure PLA finally outperforms it [33]. Based on the literature review, unfortunately, no clear information on the decomposition of the envifill^®^ M30 biopolymer could be found. Therefore, it should be assumed, in accordance with the general information, that it may take up to six months or longer for biopolymers to completely biodegrade, and certain parameters such as temperature, pressure, humidity, and the presence of particular bacteria may be necessary [34]. The bark contains a naturally occurring polyester called suberin, which is frequently depolymerized to produce suberinic acids beneficial in biopolymer applications. Particleboards can benefit from using suberin monomers, lignin, cellulose, and esters—the residue left over from the extraction of suberic acid—as filler, providing that the concentration is correct. Particleboards may also be made using the residue left over following suberinic acid extraction as an adhesive. Ethanol is the best solvent for depolymerization, which maximizes the mechanical properties of the boards [35]. Applying suberinic acids (SA) as a binder during the plywood manufacturing process resulted in a noteworthy enhancement of the plywood samples’ shear strength, suggesting a favorable impact on their biodegradability [36].

Thanks to its unique properties, suberic acid is becoming increasingly popular, not only in the cosmetics industry, but also in the wood industry. Suberin, a protective biopolymer found in plant cell walls, includes suberic acid, which helps the plant fight off infections and environmental stress [37,38,39]. Biopolymers, such as collagen and chitosan, can be employed in biomedical applications because suberic acid is used as a crosslinker to improve their mechanical and thermal characteristics [40]. It behaves polymorphically, creating many crystal forms under various circumstances that are kept stable by hydrogen bonding [41].

The study and modification of biopolymers is a vast and intricate field, offering significant opportunities and challenges. Recently, it has garnered increasing attention due to the rising demand for materials with minimal environmental impact. Biopolymers have great potential to reduce dependence on traditional plastics and contribute to more sustainable solutions, making this subject particularly relevant in environmental and material science. Notably, suberic acid, mainly its residues found in tailings, presents promising benefits. Incorporating these residues into biopolymers is expected to enhance their properties. This article explores the characteristics of PLA and modified thermoplastic starch biopolymers and examines how modifications using tree bark waste—generated during suberic acid extraction—can improve these materials’ mechanical and environmental performance.

## 2. Materials and Methods

### 2.1. Materials and Characterization

Two types of biopolymers were used for the study: polylactic acid (PLA), laboratory-purpose (Sigma-Aldrich, Saint Louis, MO, USA, product no. 38534), and envifill^®^ M30, a biodegradable thermoplastic starch used for injection molding applications. The materials are made from renewable raw materials of natural origin, biodegradable, and compostable (under natural conditions, in the presence of microorganisms), Grupa Azoty S.A. 8 E. Kwiatkowskiego St., 33-101 Tarnów, Poland. To both of these materials, 10 parts of powdered suberinic acid residues (SAR) were added by weight (Latvian State Institute of Wood Chemistry, Riga, Latvia), which have been described in detail by Makars et al. [35]. The assumed SAR addition, 10 parts by weight, is based on our own research, where the mentioned addition led to the optimal results of the tested composites [36]. The fundamental chemical characteristics of the SAR are as follows: 9.0 weight percent cellulose, 21.4 weight percent lignin, aromatic suberin, 17.5% ω-hydroxy acids, and 11.9% α, ω-diacids. Beech (*Fagus sylvatica* L.) and birch (*Betula alba* L.) wood were used to elaborate the basic mechanical parameters of the tested blends applied as a potential wood binder. The bonded wood samples have were prepared according to [42].

Fourier transform infrared spectroscopy (ATR-FTIR) was conducted using the Nicolet iS10 spectrometer (Thermo Fisher Scientific Inc., Waltham, MA, USA), where the spectrum was analyzed in the range of 4000–650 cm^−1^, recorded as an average of 16 measurements. Thermogravimetric analysis (TG) was performed using the Q500 thermogravimetric analyzer (TA Instruments, New Castle, DE, USA) in a nitrogen atmosphere, testing the samples in a temperature range from 25 to 1000 °C with a heating rate of 10 °C min^−1^. The thermogravimetric study determined the mass loss temperatures (T5%, T50%, T95%) and the percentage mass loss (Δm) at various stages of degradation. Differential scanning calorimetry (DSC) was conducted using the Q200 calorimeter (TA Instruments, New Castle, DE, USA), recording three curves: first heating, cooling, and second heating, with an analysis of the glass transition temperature (Tg), cold crystallization (Tcc), and melting (Tm). Dynamic mechanical analysis (DMA) was carried out using the Q800 analyzer (TA Instruments, New Castle, DE, USA) in a three-point bending mode, testing the samples in the temperature range of 30–140 °C and recording the storage modulus (E) at different temperatures. The density profiles of the tested panels were measured on a GreCon DAX 5000 device (Fagus-GreCon Greten GmbH and Co. KG, Alfeld/Hannover, Germany). The modulus of elasticity (MOE) and the tensile strength perpendicular to the sample’s plane (internal bond, IB) were determined according to EN 310 [43] and EN 319 [44], respectively, with the use of a computer-controlled universal testing machine (Research and Development Centre for Wood-Based Panels Sp. z o.o., Czarna Woda, Poland). A Quanta 200 (FEI, Hillsboro, OR, USA) scanning electron microscope was used to define the surface morphology of the produced biopolymer blends.

### 2.2. Preparation of Blends

The filaments with the appropriate formulation (PLA with SAR and M30 starch with SAR) were produced using the Leistritz Extrusionstechnik GmbH, Nürnberg, Germany, extruder, where the temperatures in individual extruder sections were 170–180 °C. The obtained continuous composite web was ground on a hammer mill.

The research methodology included the preparation of samples using the Argenta AW03 vulcanizing press (Argenta, Brzeziny, Poland), in which the test material in powder form was pressed at a temperature of 120 °C into plates with dimensions of 60 mm × 60 mm × 1.5 mm, under a pressure of 0.7 MPa for 30 s.

### 2.3. Statistical Analyses

Analysis of variance (ANOVA) and t-test calculations were used to test (α = 0.05) for significant differences between factors and levels using the IBM SPSS statistic base (IBM, SPSS 20, Armonk, NY, USA). The means of MOE and IB were compared, and the homogenous and non-homogenous groups were indicated in the MOE and IB results. Where applicable, the mean values of the investigated features and the standard deviation indicated as error bars are presented on the plots as error bars.

## 3. Results

### 3.1. Fourier Transform Infrared Spectroscopy 

**Figure 1:**
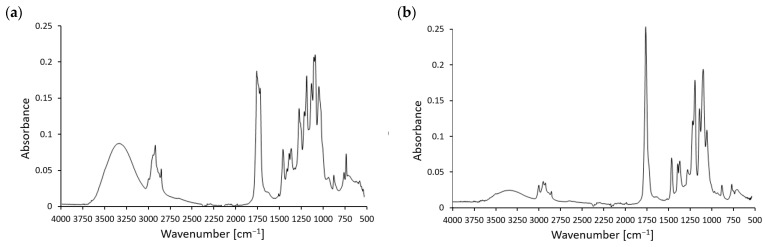
FTIR spectra of (**a**) M30 + 10% SAR and (**b**) PLA + 10% SAR samples.

Figure 1 shows the FTIR spectra of the tested materials with the addition of 10% of SAR. The difference between the spectra of the tested materials is noticeable in the range of 3750–3000 cm^−1^. In the case of the M30 material, a high-intensity peak was recorded, which was associated with a large number of –OH groups in the TPS molecule. A significantly higher intensity was also observed at the 2921 cm^−1^ peak, corresponding to the stretching vibrations of the –CH functional group. The rest of the FTIR spectrum for both materials is similar, and in the case of the M30 material, it originates from the PLA component of the mixture. Thus, the recorded peaks of both materials include a peak around 175 cm^−1^ corresponding to the stretching vibrations of the carbonyl group C=O; the appearance of a band at approximately 1454 cm^−1^ corresponding to the –CH_3_ group; and –CH deformation and asymmetric bands appear at around 1382 cm^−1^ and 1360 cm^−1^. Stretching peaks of the ester group C–O–C appear at 1266 cm^−1^ and 1182 cm^−1^, and an asymmetric C–O–C peak appears at 1087 cm^−1^. At 868 cm^−1^ and 755 cm^−1^, two bands that can be attributed to PLA’s amorphous and crystalline phases appear. In the FTIR spectrum, the OH groups show distinctive vibrational bands, usually in the 3600–3000 cm^−1^ region. The surroundings and interactions of the OH groups have an impact on these bands. For instance, the notable spectrum changes in the OH vibration range in nanocomposites made of starch and polyvinyl alcohol show interactions and miscibility between the constituents [45]. Hydrogen bonds that OH groups form can impact a biopolymer’s mechanical and thermal characteristics. For example, changes in vibrational band 1 in the FTIR spectra show that aliphatic hydroxyl groups in lignin establish stronger hydrogen bonds than phenolic hydroxyl groups. Complex spectrum patterns can result from this hydrogen bonding, particularly in biopolymers that include many hydroxyl groups [46]. 

### 3.2. Thermogravimetric Analysis 

**Figure 2:**
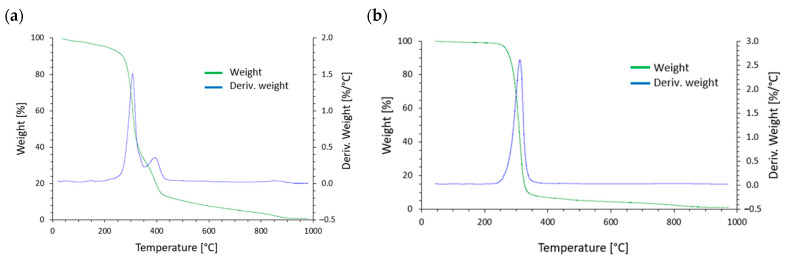
TG curve of (**a**) M30 + 10% SAR and (**b**) PLA + 10% SAR samples.

Figure 2 and Table 1 show the thermogravimetric analysis (TG) results for the PLA and M30 with the addition of 10% of SAR. The thermal resistance of the M30 material determined in the study, defined as the temperature at which a 5% mass loss of the sample occurs (T5%), was 207.2 °C. The M30 material exhibited a multi-stage thermal decomposition process, during which four distinct stages of degradation were recorded. Mass loss was observed at low temperatures. The first stage of degradation occurred in the temperature range of 20 to 235 °C, with a mass loss of 6.6%. The primary degradation of the tested material occurred mainly in the second and third stages. The second stage of degradation occurred in the temperature range of 235 to 350 °C, with a recorded mass loss of 60.6%. The degradation process in the second stage was most intense at 306.9 °C. The third stage of degradation was significantly smaller, with a recorded mass loss of 22.2% of the original sample mass. This mass loss occurred in the 350 to 445 °C range, with a maximum of 394.1 °C. Lastly, the fourth stage of degradation was much smaller. The mass loss was 9.7%, occurring over a broad temperature range from 445 to 1000 °C, with the maximum mass loss at 853.2 °C. During the TG analysis, the sample underwent almost complete degradation, as the residue after the test was minimal, amounting to only 0.9%.

A distinctly different degradation process was observed for the PLA material. One main stage of decomposition was recorded in the range from 215 to 450 °C. This stage accounted for the loss of 92.2% of the original sample mass and was most intense at 306.7 °C. The curve also showed a second, much less intense degradation stage, occurring in the 400 to 1000 °C range, with a mass loss of 6.1% in this stage. Similarly, in the case of the PLA material, the residue after the test was minimal (1.2%), indicating almost complete degradation of the tested material.

In different research, the thermal degradation behavior of PLA was compared to that of other polymers, such as polypropylene (PP) and polyethylene (LDPE and HDPE), in inert circumstances. Dynamic thermogravimetric analysis (TGA) was employed in this work to emphasize the dispersed activation energy at different deterioration phases throughout a temperature range of 303–973 K [47]. Fourier transform infrared spectroscopy (TGA-FTIR) in conjunction with thermogravimetry has been used to study the thermal degradation of PLA. The breakdown products are carbon monoxide, carbon dioxide, lactide, and acetaldehyde. Notably, carbon dioxide and acetaldehyde last until the conclusion of the trials, whereas chain homolysis causes carbon monoxide to drop beyond the peak temperature [48]. One research study identified unique degradation mechanisms below and above 330 °C, affected by leftover zinc compounds from the synthesis process, even though particular degradation peaks at 306.7 °C are not explicitly addressed. This implies that the presence of catalysts or contaminants can substantially impact degradation behavior [49].

The thermal stability and multi-stage breakdown processes of biopolymer blends like M30 have been the subject of several investigations. Blends of PLA and PHB show intricate thermal degradation behavior. When PLA and PHB are combined, PLA’s thermal stability declines while PHB’s stability increases. The kind and location of additives impact the PLA/PHB blend’s degradation process, which can significantly impact the blend’s thermal stability [50]. Polymer blends can degrade at high temperatures in a complicated way, with interactions between various polymers influencing the overall stability. For example, adding oxide ceramic powder to a polymer blend might delay degradation initially but speed it up later [51]. The relatively low thermal stability of M30 starch can be attributed to its inherent molecular structure and composition [52]. As a starch-based biopolymer, M30 is composed primarily of polysaccharides, which consist of long chains of glucose molecules [53]. These molecular structures are held together primarily by hydrogen bonds, which are relatively weak compared to the covalent bonds found in synthetic polymers. As a result, starch-based materials like M30 begin to degrade or soften at lower temperatures because hydrogen bonds break down more readily when exposed to heat.

### 3.3. Differential Scanning Calorimetry Analysis

**Figure 3:**
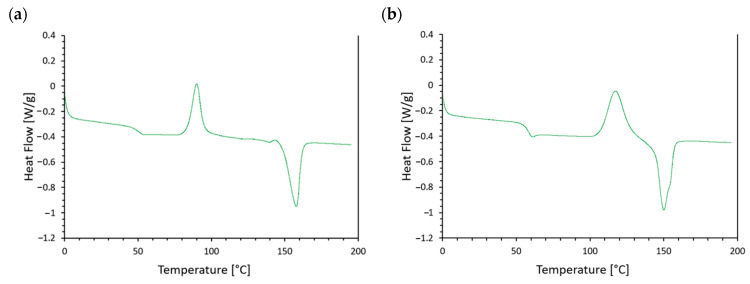
DSC curve of the (**a**) M30 + 10% SAR and (**b**) PLA + 10% SAR samples.

Figure 3 shows the DMA curve of tested samples; the values are shown in Table 2. The thermal curves of both tested materials were similar. In both cases, changes related to the glass transition, cold crystallization, and melting were observed on the recorded curves. 

The glass transition temperature (Tg) of the M30 material was 50.7 °C. The cold crystallization process occurred in the temperature range from 75 to 105°C, with the peak maximum (Tcc) at 90.9 °C. The melting process of the crystalline phase of the M30 material occurred in the range from 145 to 170 °C, with the peak maximum (Tm) at 159.0 °C.

The PLA + 10% SAR material had a higher glass transition temperature (Tg) of 59.1 °C. The cold crystallization and melting processes also occurred at higher temperatures. Cold crystallization of PLA + 10% SAR occurred in the temperature range from 100 to 135 °C, and the melting process was from 135 to 165 °C. The peak maximum temperatures for these two transitions were 118.3 °C and 151.0 °C, respectively.

The differences in the DSC curve profiles may be attributed to the plasticizing effect of thermoplastic starch in the M30 + 10% SAR material, a blend of TPS and PLA. The thermoplastic starch penetrates between the PLA macromolecules, increasing their distance and facilitating their movement. As a result, the glass transition temperature is lowered, and the cold crystallization process is accelerated.

Based on the obtained values of ΔHcc and ΔHm, it can be concluded that both tested materials are amorphous. The similar values of these parameters indicate that the entire crystalline phase, which melts, was formed during cold crystallization. It was not present in the original material, concluding that the tested materials have an amorphous (non-crystalline) nature.

Higher heating rates can increase the measured Tg values for starch and starch/gluten blends during differential scanning calorimetry (DSC). This indicates that the measurement technique can also affect the Tg results [54]. The number of recycling cycles hurts the glass transition temperature (Tg) and melting temperature (Tm) of the PLA/TPS blends, according to differential scanning calorimetry (DSC) data, showing alterations in the amorphous areas [55]. Furthermore, the PLA’s cold crystallization and melting behavior in blends imply that the processing conditions and blend composition significantly impact the amorphous regions [56].

### 3.4. Dynamic Thermomechanical Analysis

**Figure 4:**
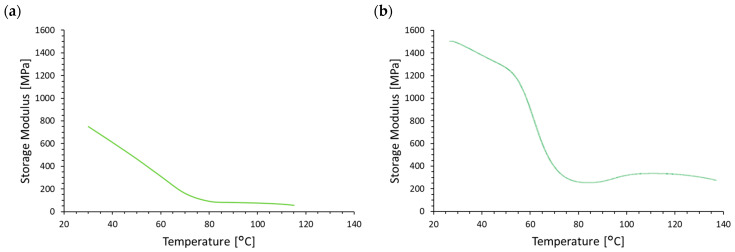
DMA curve of (**a**) M30 + 10% SAR and (**b**) PLA + 10% SAR samples.

Dynamic thermomechanical analysis is presented in Figure 4. A significant decrease in the storage modulus (E′) was observed for both materials tested between 30 and 80 °C. The E’ value of the M30 + 10% SAR material dropped from 733.6 MPa at 30 °C to 91.4 MPa at 120 °C. For the PLA + 10% SAR material, a decrease from 1491 MPa at 30 °C to 340 MPa at 120 °C was recorded.

The nature of the E’ change appears milder for the M30 + 10% SAR material, likely due to a cold crystallization process at lower temperatures, than the PLA material. The crystallites formed during cold crystallization are characterized by higher E’ values than in the amorphous phase, which “softens” the decrease in storage modulus. The effect of cold crystallization is visible in the PLA + 10% SAR material. The process occurs at a higher temperature, leading to the observed increase in E′ above 90 °C. The E’ value at 120 °C is significantly higher than at 80 °C due to the crystallites formed during cold crystallization. The strain rate during deformation determines the crystallinity and molecular orientation of poly(l-lactic acid) (PLA). Elevated strain rates increase crystallinity and orientation, which impacts the material’s mechanical characteristics and deformation behavior [57]. Anisotropy and elastic characteristics of crystalline materials, particularly biopolymers, affect their mechanical characteristics [58]. 

### 3.5. Scanning Electron Microscope Images of Blend Tested

**Figure 5:**
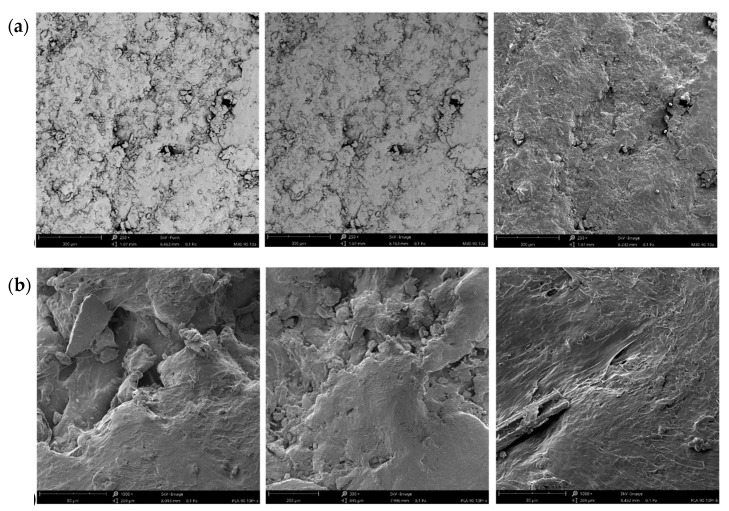
Scanning electron microscope images of biopolymer blends of (**a**) M30 + 10% SAR and (**b**) PLA + 10% SAR.

The images in Figure 5a show the surface of the M30 biopolymer with 10% SAR added at a magnification of 250×. The scale on all images is 300 µm. The images are very similar, revealing the biopolymer’s irregular, porous surface structure. In the first and second images, numerous micropores and structures of varying sizes and shapes are visible, suggesting a heterogeneous surface morphology. The third image shows a less porous surface, which appears smoother, with visible small particles embedded on the surface. This area of the biopolymer may differ in terms of treatment or processing. The addition of SAR may have contributed to increased porosity or surface irregularities. The micropores could result from interactions between the biopolymer molecules and the SAR, leading to phase separation or modification of the morphology during the forming process. In the third image, small particles are scattered on the smooth surface. These may be SAR deposits that were not fully integrated into the polymer matrix or the result of interactions between the SAR and the polymer surface, which could have influenced its distribution. The addition of SAR may affect the degree of surface homogeneity. The images reveal areas with varying levels of roughness, which may be due to the uneven distribution of SAR within the biopolymer matrix.

The images in Figure 5a show the surface of the M30 with 10% SAR added. The parameters are the same as those for the pictures in Figure 5b for PLA. The visible structures suggest that the addition of SAR significantly affects the micromorphology of the material. The surface of the PLA with SAR added has an irregular, porous structure. Small cracks or depressions are visible in some areas, which may suggest increased roughness or sensitivity to mechanical stresses. Small agglomerates are also visible on the surface, indicating the presence of the SAR modifier in the material. These agglomerates are unevenly dispersed, which may indicate local accumulation of SAR, affecting the mechanical and thermal properties of the composite. Delicate fibrous structures can be observed in the images, suggesting that the forming process of the modified PLA may have favored the formation of oriented polymer structures, which could influence the material’s mechanical strength. Differences in surface smoothness compared to pure PLA can be noted. The addition of SAR likely affected the crystallization or curing process of the polymer, leading to a rougher surface.

Biopolymer structures can have their size and form altered by fillers. For example, inorganic fillers are usually spherical and considerably smaller than organic fillers, frequently appearing as uneven rods [59]; by observing the images, you can see that we are dealing with organic fillers. Based on the microstructural changes induced by the fillers, they can be related to the observable properties of biopolymer composites [60]. Based on this information, it can be concluded that the observed changes are consistent with literature data. 

### 3.6. Density Profile as Wood Penetration Measurement

**Figure 6:**
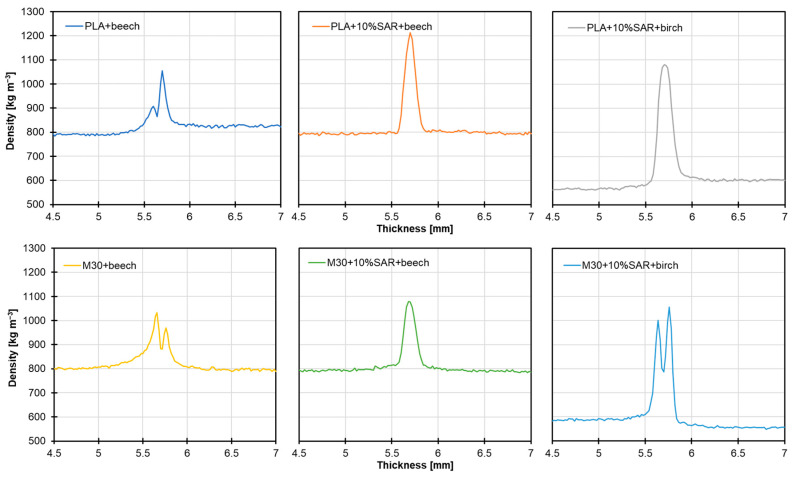
Density profiles of the tested blends as a binder of the different wood species.

Figure 6 illustrates the density profiles of polymer blends compared to various wood species. The highest joint density was observed in the PLA sample with 10% SAR, reaching approximately 1200 kg m^−3^ in the sample paired with beech wood. Birch wood’s joint density was slightly lower, around 1100 kg m^−3^. Similarly, the M30 biopolymer with 10% SAR exhibited peak bond density values comparable to the PLA/SAR blend, ranging from 1000 to 1100 kg m^−3^.

Interestingly, the biopolymer with 10% SAR demonstrated less extensive spreading than its pure form, resulting in a thinner bond and, thus, higher density than the reference samples.

A slight difference in the biopolymer’s penetration into the wood can be observed, which may be attributed to variations in the density and porosity of the wood species used. The greater the porosity, the deeper the adhesive penetrates the wood [61,62]. The degree to which adhesives may penetrate wood largely depends on its cellular structure, including the number and location of tracheids and vessels [63].

### 3.7. Modulus of Elasticity 

**Figure 7:**
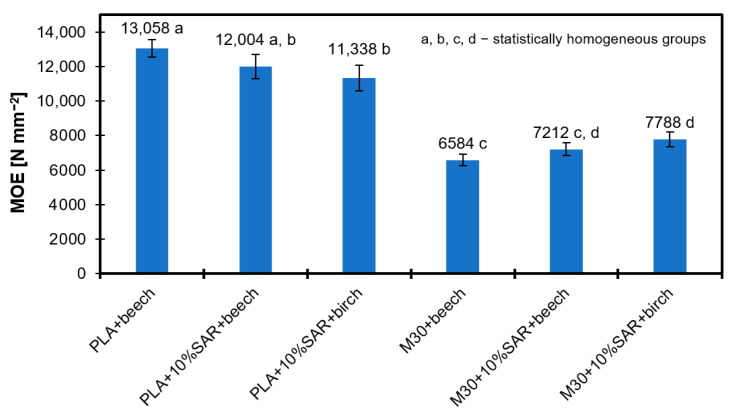
Modulus of Elasticity of Tested Samples.

Figure 7 illustrates the results of the Modulus of Elasticity (MOE) for the tested samples. The highest MOE values were recorded for the beech wood and PLA biopolymer, achieving a peak of 13,058 N mm^−2^. Incorporating the 10% SAR reduced the MOE, with beech wood decreasing to 12,004 N mm^−2^ and birch wood to 11,338 N mm^−2^. Notably, combining the M30 biopolymer with beech wood yielded the lowest MOE at 6584 N mm^−2^; however, adding the 10% SAR led to an improvement, increasing the MOE to 7212 N mm^−2^ for beech wood. The highest MOE value was recorded for the birch wood sample containing 10% SAR, which reached 7788 N mm^−2^. In summary, adding SAR proved effective as a filler in the M30 biopolymer, enhancing the MOE value. In the case of PLA, the addition of SAR decreases the MOE value, but the reductions are not drastic. Fillers can enhance biopolymers’ mechanical characteristics. For instance, natural fillers like onion peels and buckwheat groats can be added to PLA composites to improve their elongation at break and crystallinity. Still, a more extensive filler content might also decrease other mechanical qualities [64]. In the case of birch wood, a biopolymer combined with a SAR additive worked better. The porosity nature of wood, which is mainly made of cellulose, hemicellulose, and lignin, affects how it interacts with biopolymers and may contribute [65,66]. 

### 3.8. Internal Bond

**Figure 8:**
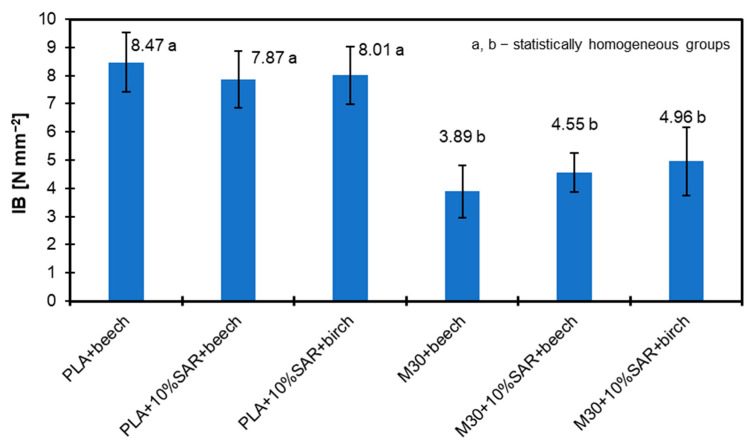
Internal Bond of tested samples.

Figure 8 presents the results of the Internal Bond (IB) tests for the samples analyzed. The trend is similar to that observed in the MOE analysis—specifically, for PLA, with the addition of 10% SAR, the IB values decrease slightly. The lowest result was recorded for the beech wood sample at 7.87 N mm^−2^. In the case of the M30 biopolymer with the addition of SAR, the highest IB values were noted for the beech wood sample, reaching 4.96 N mm^−2^.

Various factors affecting the quality of bonding of biopolymers to wood have been described in the literature: the bond-line thickness and PLA migration into the wood matrix are influenced by the processing parameters and procedure, including pressing temperature and time. In general, PLA penetration and bond strength are enhanced by higher pressing temperatures and longer durations [67]. Enhancing the interfacial adhesion between the PLA and wood by surface treatments, such as silane coupling agents, improves the composite’s mechanical characteristics [68]. Another factor is the quantity of binder utilized. While adding too much binder may not result in additional benefits, increasing the amount can enhance mechanical qualities [69]. 

Several vital factors suggest significant potential for enhancing this additive in biopolymer materials. Studies indicate that the benefits of the SAR additive to biopolymers are already evident in the variations in wood species. It is especially worth highlighting that adding 10% SAR to M30 starch leads to a significant increase in IB, depending on bonded wood species, a 17 and 28% raise, for beech and birch wood, respectively. This positive effect of SAR addition to the biopolymer blend may significantly contribute to the layered lignocellulosic biopolymer composite properties described in [70] or to the fibrous lignocellulosic composites [69].

Current research sees great potential for using biopolymer blends with SAR, mainly in the wood coatings industry, due to better barrier properties: adding SAR to PLA significantly improves gas barrier properties, lowering formaldehyde and total volatile organic compound (TVOC) emissions. For this reason, PLA/SAR composites can be used in industries such as furniture, which require minimal emissions. SAR improves the mechanical properties of PLA, including relative hardness and scratch resistance. Surface finishing products benefit from this enhancement, which provides increased protection and durability [71,72]. The solid mechanical properties and processability of PLA nanocomposites make them suitable for use in automotive components, especially when combined with SAR. These properties can be further enhanced by adding SAR, making PLA a competitive alternative to traditional materials [73]. By combining SAR’s improved barrier properties with PLA’s biodegradability, PLA/SAR composites can be used in packaging applications. This combination can help create environmentally friendly packaging options [74]. Due to the great similarity of M30 to PLA, the application area for M30 and SAR blends will converge.

## 4. Conclusions

Based on the research conducted on the PLA and M30 materials, which included the addition of 10 parts by weight of powdered suberic acid residues, the following conclusions can be drawn:

The FTIR spectra of the M30 and PLA materials display characteristic peaks for these polymers, affirming their respective identities. The M30 material demonstrates a multi-step degradation process with a thermal resistance up to 207.2 °C, a glass transition temperature of 50.7 °C, and a melting temperature of 159.0 °C, confirming its amorphous nature. At 30 °C, M30’s storage modulus is measured at 733.6 MPa, which then rapidly decreases between 30 and 80 °C, reaching a minimum of 91.5 MPa at 120 °C. In comparison, the PLA material exhibits a two-step degradation process with a thermal resistance reaching 263.1 °C, a glass transition temperature of 59.1 °C, and a melting temperature of 151.0 °C, also indicating its amorphous state. The storage modulus for PLA at 30 °C is 1491.0 MPa, decreasing swiftly between 30 and 80 °C to a minimum of 276.7 MPa at 80 °C.

In terms of mechanical properties, the highest MOE values were recorded for the beech wood and pure PLA biopolymer (13,058 N mm^−2^). However, adding 10% SAR led to a reduction in MOE for both beech wood (12,004 N mm^−2^) and birch wood (11,338 N mm^−2^). The combination of the M30 biopolymer with 10% SAR improved MOE for beech wood to 7212 N mm^−2^, with birch wood achieving a maximum MOE of 7788 N mm^−2^ with the same SAR addition. A similar trend was observed in IB values, where the lowest IB of 7.87 N mm^−2^ was recorded for beech wood, and the highest IB of 4.96 N mm^−2^ for the M30 biopolymer with SAR. These findings highlight significant potential for further enhancements in SAR-modified biopolymer materials across various wood species.

The research confirms the potential of the SAR additive in biopolymers. The effectiveness of this solution depends not only on the biopolymer used but also on other variables that can enhance the parameters examined during wood bonding. It is important to emphasize that this is a biodegradable solution aligned with circular economy principles due to the presence of SAR. While the M30 and PLA blends with added SAR exhibit some favorable properties, several key factors limit their practical applications. Although they offer relatively high thermal resistance, both M30 and PLA are susceptible to degradation at elevated temperatures, with significant softening occurring at 207.2 °C for M30 and 263.1 °C for PLA. This limits their suitability for high-temperature applications. The mechanical performance of SAR-modified materials also poses a challenge; while there is some improvement in the MOE for the SAR-modified M30, it remains significantly lower than pure PLA, which restricts these materials in structural applications that require high rigidity. Furthermore, the amorphous nature of both M30 and PLA reduces their durability and resistance to physical wear, making them less suited for demanding conditions involving abrasion or long-term mechanical stress. A notable decrease in storage modulus at temperatures between 30 and 80 °C raises additional concerns about stability, as this rapid softening compromises the material integrity in applications needing consistent mechanical properties. Finally, challenges in processing and scaling due to the need for precise control over SAR dispersion add technical and cost barriers, making large-scale industrial applications difficult to achieve.

## Figures and Tables

**Table 1:** Thermogravimetric test results.

Sample	T_5%_ [°C]	T_50%_ [°C]	T_95%_ [°C]	T_max1_ [°C]	T_max2_ [°C]	T_max3_ [°C]	Δm_1_ [%]	Δm_2_ [%]	Δm_3_ [%]	Δm_4_ [%]	R [%]
M30 + 10% SAR	207.2	314.5	728.2	306.9	394.1	853.2	6.6	60.6	22.2	9.7	0.9
PLA + 10% SAR	263.1	303.1	551.9	306.7	-	805.6	92.2	-	-	6.1	1.2

**Table 2:** Differential scanning calorimetry test results.

Sample	T_g_ [°C]	T_cc_ [°C]	ΔH_cc_ [J/g]	T_m_ [°C]	ΔH_m_ [J/g]
M30 + 10% SAR	50.7	90.9	17.2	159.0	21.37
PLA + 10% SAR	59.1	118.3	29.6	151.0	29.7

## Data Availability

https://doi.org/10.18150/4SFTST (created and accessed on 21 October 2024).
